# Television documentary, history and memory. An analysis of Sergio Zavoli's *The Gardens of Abel*

**DOI:** 10.1080/1354571X.2014.962258

**Published:** 2014-11-04

**Authors:** John Foot

**Affiliations:** ^a^ University of Bristol

**Keywords:** Documentary, Italian television, Sergio Zavoli, Franco Basaglia, psychiatry

## Abstract

This article examines a celebrated documentary made for Italian state TV in 1968 and transmitted in 1969 to an audience of millions. The programme – *The Gardens of Abel* – looked at changes introduced by the radical psychiatrist Franco Basaglia in an asylum in the north-east of Italy (Gorizia). The article examines the content of this programme for the first time, questions some of the claims that have been made for it, and outlines the sources used by the director, Sergio Zavoli. The article argues that the film was as much an expression of Zavoli's vision and ideas as it was linked to those of Franco Basaglia himself. Finally, the article highlights the way that this programme has become part of historical discourse and popular memory.

## A revolution in psychiatry and a TV documentary

In the 1960s, a small group of radical psychiatrists in Italy began to transform mental health care. The focus of the early movement was the town of Gorizia, in the extreme north-east of the country, right on the border with Yugoslavia, where a psychiatrist called Franco Basaglia took over as Director of the Provincial Psychiatric Hospital in 1961. In the years that followed he revolutionized the institution of which he was in charge – altering the architecture of containment, freeing up the patients and empowering them. Daily general assemblies were introduced and the patients produced their own newspaper. Basaglia was influenced by a mixture of theories and practical examples of reform – from phenomenology to the Therapeutic Communities movement in the UK to his reading of Primo Levi's work on his time in Auschwitz. He often compared psychiatric hospitals to concentration camps (Bucciantini 2011, 69–91).

Basaglia (and the group he worked with) began to publicize their ideas and produced a best-selling collective book – *L'istituzione negata: rapporto da un ospedale psichiatrico* (*The Institution Denied. Report from a Psychiatric Hospital*) – in 1968 (Colucci and Di Vittorio 1987; Legrand 1988; Depardon 1982; Scheper-Hughes and Lovell 1987; Ramon and Giannichedda 1988; Donnelly 1992; Giannichedda 2009; Slavich n.d.; Grubissa 2011). The hospital soon became one of the key sites of the 1968 movement. Volunteers, militants and journalists flocked to Gorizia to see what was going on there and participate in the reform process. In that same year, a well-known TV director, Sergio Zavoli, shot a documentary in Gorizia – which he called *I giardini di Abele* (*The* *Gardens of Abel*).^1^

This article examines the content of this programme in detail for the first time, and questions some of the claims that have been made for it in terms of the evidence, as well as outlining and analysing the sources used by the director. The article argues that the documentary was as much a product of Zavoli's own personal vision of society as it was a reflection of Basaglia's ideas about institutions and mental health. Finally, the article highlights the way that this programme has become part of historical discourse, underlining the importance of this medium in popular memory. Zavoli was a left-wing Catholic (he was sometimes called ‘God's socialist’) and had worked in radio for many years, producing award-winning sound-only documentaries such as a study of a covent (*Clausura* 1958), which was translated into six languages, before moving to the TV sector. For the TV critic and historian Aldo Grasso, this was a documentary ‘which was heard all around the world’ (1996, 855). Zavoli had become famous in the 1960s thanks to his innovative programme *Il Processo alla Tappa (The Trial of the Stage)*, which covered Italy's annual cycling race, the Giro d'Italia (Grasso 1996, 855; Spadaro 2012; Zavoli 1969b, 2002; Foot 2011, 202, 308).

Zavoli and his team spent some time in Gorizia in 1967/68, filming and preparing the programme and discussing its content with Basaglia and others. There are indications that some of those working on the programme were also attending patient and doctor-led meetings in 1967 or even earlier. He also collected film material shot by an amateur film-maker from the early 1960s – showing patients in the courtyard of the hospital and also patients pulling down a perimeter fence in a ‘moment of liberation’ orchestrated by Basaglia. Zavoli pieced together this material with various extracts from archive film of other hospitals and previous documentaries. None of these other sources was credited in the version of the documentary shown on national television in 1969. A debate amongst nurses was staged for the documentary. Patients were shot leaving the hospital, going for walks and wandering in the gardens, and Basaglia was interviewed in his office. Finally, Zavoli interviewed a number of patients. Extracts from four of these interviews made the final cut of the documentary.

It is also clear from my research that Zavoli was given material by Basaglia as background to the film. In particular, the preperatory images and text for a celebrated and much-cited photobook called *Morire di classe* (*To Die Because of Your Class*), edited by Franco Basaglia and his wife Franca, were crucial to the content and structure of the film. The book would appear in print in 1969, after Zavoli's programme had gone out on air (Basaglia and Ongaro 1969).

This article shows that there were strong parallels and connections between the thrust of Zavoli's programme and that photobook. Quotations and arguments from *I giardini di Abele*, and some of the images shown as stills, were taken directly from that volume. These two texts should therefore be analysed together. Quotations and ideas from that book were incorporated into Zavoli's voiceover and one image from the book was used in the documentary. In addition, numerous citations from that book (citations were used alongside photo images) were picked up and adapted. Thus far, however, despite frequent references to Zavoli's film in the secondary literature, no critic or historian has commented on or even noticed the links between *Morire di classe* and *I giardini di Abele*.

One of my arguments in this article is that the documentary that emerged from Zavoli's time in Gorizia was a blend of Basaglia's ideas and Zavoli's interpretation of what he had seen and heard there. Basaglia had become one of the leaders of the critical or radical psychiatry movement at that time (which was also often called ‘anti-psychiatry’) – so much so that the movement was often described as ‘Basaglian’. While the film clearly supported the changes introduced by Basaglia in Gorizia, and gave ample space to Basaglia's philosophy in an interview with him included in the documentary, the overall feel of the film was also very much linked to Zavoli's religious and political outlook – and his desire to tell a ‘universal’ and ‘poetic’ story. This was not a story that Basaglia was particularly interested in, however. Zavoli used Basaglia for his own ends, while Basaglia – at the same time – was happy for Zavoli to publicize the changes happening in Gorizia and elsewhere. The differences between the two men in terms of their ideas and outlooks have rarely, if ever, aroused any comment.

Crucially, Zavoli wanted to draw attention to what he saw as the ways in which the existence of the mentally ill was a problem for *everyone* – at a high, spiritual level. Zavoli was interested in a collective ‘conscience’ concerning these people (the ‘mad’) and the places where they were locked and hidden away. Basaglia, on the other hand, had a much more radical and non-spiritual approach to his work. He believed that a *social* and *political* analysis of the asylum system was required. Basaglia argued that contradictions should be ‘opened up’ within the system to help to ‘overturn’ the institution of which he was Director. Zavoli watered down Basaglia's ideas, and at one point made a clear statement in opposition to some of what Basaglia was saying. In addition, the contrast between ‘open’ and ‘closed’ hospital systems drawn by Zavoli was a moderate and reformist interpretation of what Basaglia thought he was doing. Thus, while the documentary gave space to Basaglia's ideas – and often reflected them directly – its overall feel was linked to Zavoli and his long dominant voiceover, the editing, and the heavy authorial feel imposed by the director. The radical ‘Basaglian’ terms ‘overturning’ and ‘negation’ were never used in the film, despite the existence of a best-selling book with that title. Zavoli's message was intended to be universal, while Basaglia's was political, specific and linked to daily practice and activity within the hospital. The 1969 programme can only be understood if we see it as a combination, a hybrid, of Basaglia's vision (and that of his team) *and* (above all) Zavoli.

This article now goes on to analyse the programme itself and the claims made for it over the years. I underline the fact that, while the programme is often cited, it has never been analysed or unpicked in a serious way. It is almost as if people have watched the documentary, but they haven't actually *seen* it. It has certainly not been studied. Undoubtedly, the programme marked an important moment in the history of public television and is now often seen as a kind of historical document. But we need to be very careful here. Claims for the programme cannot simply be taken at face value. Evidence and analysis are required. We also need, finally, to look at the programme itself. I now look at the documentary in detail – taking in the voiceover (written and spoken by Zavoli), the images, and the sources for the film.

## *I giardini di Abele*: analysing the documentary

‘Gorizia. I am here because of the psychiatric hospital. The madhouse – if you will.’ (Zavoli, voiceover, *I giardini di Abele*, 1969a)

Zavoli: Then, one day, the hospital was opened up. What changed?'

Patient [‘Carla’]: Everything!

(Extract from filmed interview with Carla Nardini [Patient], Gorizia, Psychiatric Hospital, 1968)

A TV camera is moving down a long flat road, mounted on a car. The place is an anonymous one. It could be anywhere; any city. A voiceover starts in the background. This was a well-known voice in Italy – a household name – that of Sergio Zavoli. It was 1968, and he was in Gorizia. The voiceover continued:^2^

We tend to find the mentally ill on the edge of our cities, perhaps because their presence worries us. Here I am in Gorizia, at the extreme periphery of the city: one wall of the hospital marks the border between the Italian state and that of Yugoslavia. I wanted to get to know about what is going on in this hospital because the history of this place has attracted the attention of scientists and men and women of culture from across the world, but in Italy this experiment risks being associated merely with a piece of bad news. (Zavoli 1969a, voiceover)

**Figure 1:**
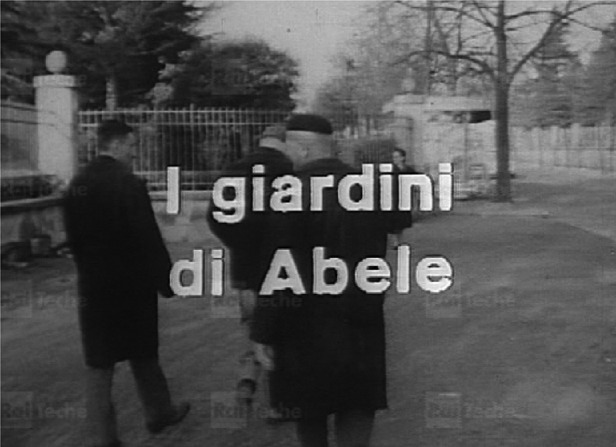
Patients leaving the hospital (title sequence). Rai-Teche.

Soon, the mounted camera turns into a side road, and breaks through the (non-existent) gates towards the asylum itself (Pitrelli 2004, 74). Soon, we shall see patients leaving via these same gates (see Figure 1). Zavoli's voiceover was autobiographical, told in the first person, although he never appears physically in the film beyond his voice. He had chosen to visit Gorizia, he says, because it had become famous, internationally. People were talking about what was happening there.

The next shot was from a photo by Carla Cerati of a man holding his head in his hands: a psychiatric patient from the asylum in Colorno. This image would be reproduced time and time again by the Basaglian movement in the years to come. It would appear soon afterwards in *Morire di classe* and had already been seen on posters and in exhibitions. Cosimo Schinaia, a historian of photography, later referred to this photo as ‘the symbol of marginalization in asylums’ (2005, 41). The voiceover also mentioned, somewhat obliquely, a recent controversy over a murder committed by a patient on day release from Gorizia's asylum. This was the so-called ‘Miklus case’ that broke at the end of September 1968 and which Zavoli referred to as a ‘*fatto di cronaca*’ (‘a piece of bad news’). A patient (Giovanni Miklus) on day release from Gorizia's asylum had murdered his wife with an axe, leading to national press coverage and a local backlash against the Gorizia experiment led by Basaglia, as well as judicial proceedings against Basaglia and others (which, in the end, came to nothing). The Miklus case had possibly led to the postponement of the screening of the film, which had been shot and edited in 1968, until early 1969.

**Figure 2:**
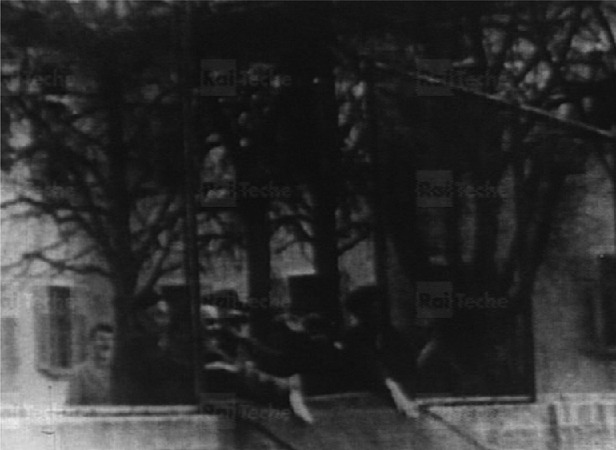
Patients knocking down the fences around the hospital, Gorizia. *Source*: Giorgio Osbat, used uncredited in *I giardini di Abele* (1969a). Rai-Teche.

This 27-minute made-for-TV documentary, directed, written and narrated by Zavoli with the help of a large crew was notable for its direct interviews with patients and with Basaglia himself. It was shot in stark black and white, and used music, a voiceover, various fragments of archive film (none of which was sourced or cited in the credits) and other visual effects including close-ups and slow motion. This was a made-for-TV film with cinematic qualities and a (it seems) a large budget. It portrayed patients leaving the hospital to go to work or on walks, and carried discussions about the meaning of madness itself. It also showed patients knocking down their own fences (see Figure 2). Some of this film was shot by a local photographer and amateur film-maker, and has recently been edited together by a film-artist and set to music (Ricci 2014). Other inmates described in their own words how they had been tortured and mistreated. One denied that he was ill at all, another cried. Most of the interviewees ‘seemed’ normal, and spoke directly to camera. It was didactic, pedagogic and educational, but also (knowingly) ‘poetic’ and rhetorical.

The Zavoli film was shot during 1968 and screened on 3 January 1969. It went out at about 9.25 p.m. on a Friday night, on RAI Uno, Italy's main TV channel, just after the news. This was a very good slot, prime time, and it was also just after Christmas, when many families would have been at home on holiday. *I giardini di Abele* was clearly a key moment in putting the Gorizia experiment on the national map. At that time there were only two TV channels available in Italy. The documentary was shown as part of a popular weekly news-documentary magazine programme called TV7, which usually contained two or three separate short films. TV7 ran on a weekly basis from 1963 to 1971. From 1967 the show aired on Friday evenings. The first film that evening in 1969 was dedicated to the new phenomenon of heart transplants. TV7 was a programme that attracted millions of viewers, a popular current affairs container slot, with high audience satisfaction levels. We do not have figures for that specific episode, but the edition on the 17 January attracted a striking 13.7 million viewers, for example, and the average viewing figure for 1969 was 11 million. Before TV7, Gorizia had been discussed in newspaper articles and books, but a mass television audience took this exposure to a whole new level.

We can safely assume, therefore, that millions of Italians saw *I giardini di Abele* in 1969. This was way above the possible readership of *L'istituzione negata*, the best-selling book produced by psychiatrists in the asylum about the changes that had taken place there in the 1960s, which sold something like 50,000 copies in the late 1960s and early 1970s. This viewing figure alone shows how important Zavoli's programme was. Basaglia, Gorizia and the patients themselves entered the living rooms of Italians across the country, from Palermo to Trieste (RAI 1969, 9–10). Millions of people were now aware of what was going on in Gorizia in 1968–1969, at least in terms of Zavoli's version of events. However, this stark figure and basic fact does not tell us anything at all about the specific effects of the Zavoli programme, or the interpretation of that programme in the minds of these viewers. Critics, historians and others would go on, in subsequent years, simply to assume and state these ‘effects’ in bold and confident terms – without any real evidence to back these statements up. Antonio Slavich, who was Basaglia's colleague and fellow reformer from 1962 to 1969 in Gorizia, argued that the Zavoli programme was the ‘real transmission belt with the rest of Italy for “Gorizia” in terms of images and ideas’ (n.d., 4).

*La Stampa's* TV review praised Zavoli's programme for its ‘deep sense of humanity’ but criticized the voiceover for being ‘over complicated and literary’ (‘Con il cuore nuovo’ 1969). TV reviewing in Italy was not as ever-present as it would become in the 1980s and 1990s, and programmes often received small or perfunctory reviews. Few critics made much of *I giardini di Abele* when it was first shown. This would change over time, although there is very little critical work available on the film itself.

As we shall see, the actual influence of Zavoli's film is simply taken as read. For example, a recent thesis concludes that Basaglia's words ‘were engraved in the hearts and minds of millions of Italians’ (Spadaro 2012, 264; Pitrelli 2004, 72). But there is little evidence available to back up statements of this kind (Foot 1999). Millions of people saw the programme – but does this mean that Basaglia's words were ‘engraved on their hearts’? What is beyond doubt is that the film has had a long afterlife. It was widely shown at the time in various venues (outside of TV itself) and continues to turn up at ‘Basaglian’ commemorations and other events. It was also re-issued on VHS as part of a series of Zavoli's films released by the RAI in the 1990s, and its script has been published in various texts published by Zavoli (and in a variety of versions). *I giardini* is very much part of the Zavoli canon of landmark TV from the 1960s and 1970s and has become part of a kind of official history of the medium. In some ways it is now part of the history of Italy itself (Crainz 2003, 249). But none of this is uncomplicated, or straightforward.

Zavoli's film has thus become a key element in the way that ‘Gorizia’ and the ‘Basaglian revolution’ is narrated and remembered. It is shown frequently, often at anniversary events, and has appeared fairly often on television. It is also available in various versions on the Internet. The film contains most of the common elements of the standard ‘Gorizia’ story, as told in other accounts: the key, charismatic role of Franco Basaglia himself, a contrast between ‘closed’ and ‘open’ institutions, a strong implied connection between mental illness and social class (which Zavoli nonetheless contested openly in the programme) and another one between mental health treatment and wealth (which Zavoli accepted); the voice given to the patients, a sense of resistance to change which needed to be overcome and aspects of horror and torture as features of the ‘old’ asylums. But Zavoli's film also added other, original and different elements: visual depictions of Gorizia and the patients and their voices, the voice and face of Basaglia himself, and a specific ‘Zavolian’ social and quasi-religious reading of what was going on in Gorizia and the issues the experiment there had thrown up.

Zavoli's programme was divided into various ‘acts’ – the smooth entry into the hospital (the prologue), the historical background (the ‘closed hospital’), the nurse's debate (which represented the idea of a struggle against the forces of conservatism), the interview with Basaglia, a voiceover analysis over film of patients in the park, interviews with the patients, and a final march towards redemption, or perhaps to nowhere (the epilogue; Spadaro 2012, 262).

             ^*^^*^^*^

One particular exchange from Zavoli's interview with Basaglia (shot in a small office, with Basaglia walking back and forth) is often repeated and cited (Pivetta 2012, 7). Zavoli asked Basaglia if he was ‘more interested in illness or in the ill’ and Basaglia replied ‘without doubt, the ill’. Basaglia, in the interview included in the film, also made clear his opposition to traditional psychiatry. When Zavoli asked Basaglia this question, ‘This hospital is accused of being more an act of civil protest than a psychiatric innovation’, Basaglia replied with ‘Absolutely. I completely agree’ (see also Forgacs 2014, 200). Thus, in 1969 Basaglia's radical ideas about mental illness were exposed to a mass audience for the first time, and these same viewers were also shown the supposed effects of the Basaglian experiment. It appeared as a significant moment for the movement, especially in retrospect. For Babini, ‘it is also thanks to that programme of the 3 January 1969 that the history of Italy and the history of psychiatry came together – a coming together which obviously had roots which went much further back in time’ (2009, 8).

Big claims are thus made for this programme, some of which are inaccurate. It is often argued that *I giardini di Abele* was ‘the first time’ that a camera had ‘entered’ an asylum and filmed patients inside the walls of a ‘total institution’ (see, e.g., Pivetta 2012, 122). However, this common claim is mistaken. A short documentary film called *La porta aperta* was shot by Michele Gandin inside Gorizia and shown in 1967. Its script/commentary is credited to Franca Ongaro, Basaglia's wife and a key activist in Gorizia. Another documentary entitled *1904, n 67* was made by Riccardo Napolitano in the same year. Moreover, as Babini shows, in 1966 a much less cited documentary was partly shot in psychiatric hospitals in Varese and Turin, and in 1961 another film-maker had also shot scenes inside an asylum for a film called *Il sistema sbagliato* (Babini 2009, 7). Zavoli also lifted a number of exact phrases and shots from the Gandin film, including the famous Osbat film of patients pulling down fences in Gorizia. The pioneering claims made for Zavoli's film are, to say the least, somewhat exaggerated.

It is true, however, that *I giardini di Abele* was the first time that patients from inside a psychiatric hospital were given something of a voice, and space to express their opinions (although this ‘voice’ was controlled and framed, clearly, by the director, the setting, the questions and the editing and Zavoli's voiceover intepretation of what had been said; Babini 2009, 8). The further difference, moreover, with Zavoli's film was the mass audience it reached (the other films were seen above all by selected audiences in local settings) and the charismatic presence of Basaglia himself.

Zavoli's film aimed to show how different Gorizia was to almost every other psychiatric hospital in Italy (and not just in Italy). Throughout the film, a sharp distinction was drawn between what he called the ‘closed asylum’ and this ‘open asylum’.^3^ The traditional approach to patients in psychiatric hospitals in televisual terms had been to ignore them altogether, and to treat them effectively as ‘non-persons’. In earlier documentaries patients were shot from behind, or without showing their faces, and doctors with white coats explained the treatments they performed on this faceless mass of ‘the ill’. They were objects, not subjects.^4^ They had certainly never been filmed as if they were real people before, or interviewed as if they could speak for themselves. The very fact of being closed inside an asylum was usually seen as something to be ashamed of. It needed hushing up. It carried huge levels of stigma. Now it was society itself that risked feeling ashamed, that was confronted with its own ‘dark side’, which had previously been hidden ‘on the edges of town’. In this sense, at least, Zavoli's programme was something very different.

## Open and closed institutions

After entering the ‘open’, ‘Basaglian’ hospital, Zavoli outlined what life was like in other asylums, and how things had been in Gorizia in the past, accompanied by archive film of patients in the courtyards of Gorizia, from the time when the hospital was ‘closed’. These clips show patients sitting, nodding repeatedly and lying down on benches. Zavoli continued:

These images were taken in this hospital. They document the reality of a closed hospital, a traditional institution. In Gorizia, that period has been rejected and filed away as part of the past – its seems now to be far away.

Zavoli was aware of the dangers of voyeurism, of the fascination-rejection that people had towards ‘the mad’, their ravaged faces, their repetitive movements. ‘We could be accused,’ Zavoli went on,

of practising new forms of violence against these people by showing without any kind of filter those faces filled with pain which are usually hidden away because of the fear of stigma which accompanies those who live in this world of the excluded, within the walls of the hospital.

Yet some sections of the film *were* undoubtedly voyeuristic: the close-ups in the interview section, the shots of patients in the final part, the focus on falling-apart shoes and twitching feet (Pitrelli 2004, 74).

Zavoli employed Basaglian and Levian language in his long narration, describing how inmates had been institutionalized. There was also a risk of humiliating the patients in filming them, but, argued Zavoli,

what we will show, quite apart from the face of the inmates and their madness, is that what remains of a man after the institution delegated to look after him has systematically objectively reduced him to a number, to a thing.

This phrase harked back to a quote so often used by the Basaglias in various articles and speeches, taken from Primo Levi's *If This is a Man*:

Imagine now a man who is deprived of everyone he loves, and at the same time of his house, his habits, his clothes, in short everything he possesses: he will be a hollow man…. He will be a man whose life or death can be easily decided with no sense of human affinity. (Levi 1997, 21, 1979, 33; Bucciantini 2011, 143)^5^

The inmates of an asylum were then compared by Zavoli to ‘blacks, the stateless, the lumpenproletariat, the Jews and as such they are often the victims of prejudice and mistreatment’. Again, this analysis was Basaglian (with strong shades of Fanon) and drew on comparisons with concentration camps but also on a social analysis of the issues at stake. This citation was very similar to that of French psychiatrists Le Guillart and Bonnafé (1952) used on both the cover of the first book to discuss the Gorizian experience edited by Basaglia (1967), *Che cos'è la psichiatria?* (*What is Psychiatry?*), and as one of the epigraphs inside *Morire di Classe* (Basaglia and Ongaro 1969):

these are ill people who cannot defend themselves, without a voice and without any rights. The mentally ill are the blacks, the indigenous populations, the Jews, the proletarians…. Like these groups, they are victims of prejudices and injustice, but these prejudices and injustices have nothing to do with the nature of madness.

Levian themes were taken up again later on, with a shot of shoes piled up which was reminiscent of similar piles of shoes in Auschwitz and elsewhere.

During this section, the viewer saw various examples of a Foucaultian-Goffmanesque ‘total institution’ (although this term was never used in the film, as Zavoli preferred the phrase ‘closed hospital’) – which described the architecture and functioning of the asylum, with locks being shut, bars, grills, a patient being held down (accompanied by dramatic music) and placed in a strait jacket, barred windows, baths in the centre of rooms, hooks with numbers on them (Goffman 1991; Foucault 2007).^6^ Many of these shots were similar to the photos that would later appear in the *Morire di classe*. There can be no doubt, as we have argued, that Zavoli consulted the proofs and preparatory material for that book, and that Basaglia suggested this material to him during their discussions in Gorizia about the film they were making. This comparison has never been drawn by any of those who have discussed this film, or indeed the book *Morire di classe*.

This montage sequence relating to the ‘closed hospital’ (which was in the main not shot by Zavoli and his team, but taken from various other sources) was then contrasted, to the sound of more hopeful and upbeat music, with archive shots of the patients themselves knocking down the fences that held them in (fences we had earlier seen in the pre-Basaglian, ‘closed’ hospital) and then original footage of people simply leaving the hospital altogether, for a walk, seemingly unaccompanied (apart from by Zavoli's camera). These were the same gates that Zavoli had entered, by car, at the start of the film. This new, reformed, hospital, it seemed, really was ‘open’, and the patients themselves had participated in this ‘opening’.

             ^*^^*^^*^

For Zavoli, throughout the film, the key issue at stake was not to do with ‘mental illness’, but with society. *Everyone* was responsible for what was going on. This was not a film aimed at psychiatrists, or patients, but at Italians in general. As Babini argues, the film was not merely a report on what was going on in Gorizia, it was also concerned with people's general attitudes to mental patients and to mental illness. It was a film about the ‘sane’ as much as it was about ‘the mad’. As Babini (2009, 8) has argued ‘Zavoli's voice (and we never see Zavoli himself) has the calm and yet firm tone linked to a moral conscience, because, like it or not, *The Gardens of Abel* also tell us about Cain.’

## An ‘overturned institution’?

It was then time for Zavoli to tell the story of what had happened in Gorizia in more detail. He provided his viewers with a short history lesson:

In November 1962 the équipe led by Franco Basaglia opened up the first wards in the Psychiatric Hospital and Italy saw its first therapeutic community. The life of the hospital was marked by ward and general meetings. The mentally ill were given, once again, a social and human role in life. They managed their own lives, thanks to ongoing communication with those who looked after them.

Once the ‘prison-like nature’ of the institution had been ‘suppressed’ by Basaglia and his équipe ‘it was time to start to understand the kind of prejudice to which the mentally ill had been subjected’ (and this is another citation that seems to pick up from Le Guillart and Bonnafé). After this historical introduction, which could have been written by Basaglia himself, and was certainly fruit of discussions with Basaglia and the équipe in 1968, we were then plunged into a meeting of nurses, some of whom were clearly opposed to the changes that had taken place in the hospital.^7^

**Figure 3:**
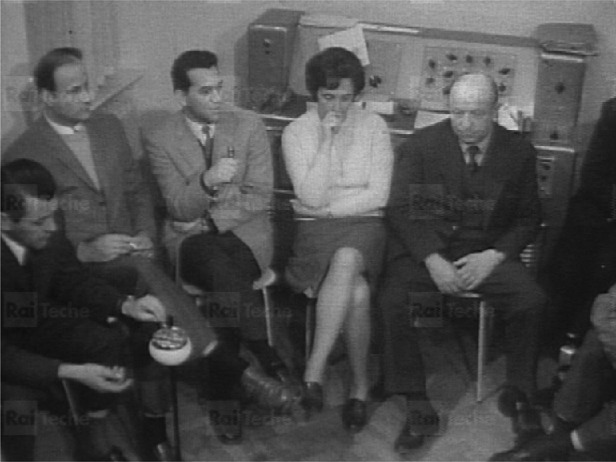
The nurse's debate. Rai-Teche.

The contrast in this constructed and somewhat artificial ‘nurse's debate’ (where all the participants held microphones, and where a large electroshock machine was clearly visible in the background; see Figure 3) was that between the idea of patients as dangerous and uncontrollable individuals, unfit to take part in normal social activities, and those with whom a relationship could be developed. The message from this debate was straightforward: change had taken place in Gorizia, but it had been in the face of opposition, and this resistance to change was still ever-present, even within the ‘open’ institution. It had not by any means been easy. A considerable number of nurses were deeply opposed to the Basaglian experiments in Gorizia. In 1968 they issued a document that complained that ‘Over the last year or so, every week, we have been subject to attack.’ The doctors, it was said, ‘have even turned the patients against us’. They particularly criticized the general meetings: ‘[W]e are unhappy with all the meetings that have been held.’ ‘These meetings,’ the nurses continued,

appear, on the surface, to demonstrate how the patients are governing their own lives – but everything is constructed, directed and pre-prepared. The same people - more or less - take part in the debates and make speeches - and act as spokespeople for the proposals of the Leader.^8^

The nurses ‘debate’ was a way of bringing these disputes out into the open.

But some nurses had been transformed by the experience, and one of these ‘Basaglian’ nurses spoke out during the filmed ‘debate’:

Here, in the hospital in Gorizia I feel different. When I meet these people [the patients] when they are out in the park, and I talk to them … you can see that they are not afraid … I don't feel like I used to; I am no longer a custodian or the prison officer that I used to be.

Interestingly, Zavoli chose not to show film of an open patient meeting (which had, it seems, been filmed by his crew).

Extracts from a general open patient meeting would not have fitted with the rest of the film. The tone of the entire documentary is controlled and elegiac, ‘poetic’ and a reflection of the directorial voice of its author, director and narrator: Zavoli himself. It is very much a film ‘by Sergio Zavoli’, heavily directed and measured, precise, authorial, sermon-like: and this creates a certain distance from many aspects of the spontaneous chaos of the Gorizian experiment. Zavoli never lost control of his own film.^9^

**Figure 4:**
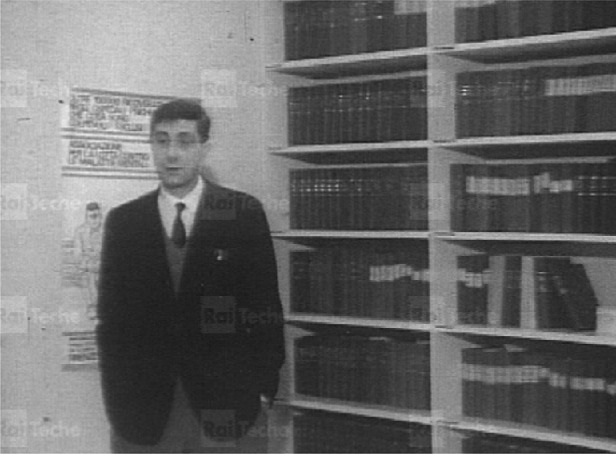
Basaglia in his office in Gorizia (addressing Zavoli, who is out of shot). Rai-Teche.

Next came the celebrated and oft-cited interview with Basaglia, who spoke as he walked quickly, up and down, in a small office. Basaglia was youthful with short hair, dressed in a jacket and tie, surrounded by scholarly tomes and papers. Zavoli remained in the background again: as just a voice, almost as if he was directing Basaglia's movements (see Figure 4). Two identical posters designed by the illustrator Hugo Pratt (a childhood friend of Basaglia's) were on the side walls, they read: ‘Oltre 100,000 ricoverati negli ospedali psichiatrici. Cosa sono? Colpevoli? Esclusi? Matti?’ [There are more than 100,000 people inside psychiatric hospitals. Who are they? Guilty? Excluded? Mad?] Basaglia introduced a stark statement into his social reading of the asylum system: ‘I do the job I do in the full knowledge that there are two kinds of psychiatry: that for the poor and that for the rich.’ He claimed that ‘nobody’ knew what mental illness was, and he talked about building relationships with patients. He also argued that the whole asylum system was bankrupt, and had nothing to do with caring for people. This was a powerful set of anti-psychiatric statements. Basaglia and his ideas were given ample space – within the confines of Zavoli's overall take on the issues at stake. Even in the Basaglia interview, the heavy hand of the director was clear. Zavoli's questions framed the interview, and the cramped room made it seems as if the camera was having difficulty keeping up with Basaglia as he strode from wall to wall.

             ^*^^*^^*^

The third act of the documentary took us into the gardens of the asylum, gardens that had been cited in the opening prologue (and in the title of the documentary itself). Classical music accompanied the camera as it swooped and floated through the gardens, in slow motion, almost hiding amongst the trees.^10^ Finally, the title of the programme was explained, with its biblical connotations.

In these vast and hospitable gardens, with their ancient beauty, we can see much of the hypocrisy with which, usually, we protect ourselves from our consciences. In these thriving gardens, on the other side of the fence, we can observe the mentally ill living their free and serene lives, but in reality these are the gardens of our inconvenient brothers – the gardens of Abel. (Zavoli 1969b, 241)

The slow motion and music was used to prick the emotions of the audience, as patients walked around the grounds of the hospital, sometimes accompanied by nurses. For Zavoli, the whole issue of mental illness and its treatment forced us to look deep into ourselves. His programme served, he thought, to highlight this contradiction, to underline the presence ‘at the edges of our cities’ of these ‘inconvenient brothers and sisters’. By observing these people, we were forced to observe ourselves.

Another short but dense history lesson followed, against the background of patients wandering in the gardens, or shots of the spectacular vegetation there. There was a direct quote from the 1904 law which had set up many psychiatric hospitals and (until 1968) was still in force, and strong hints at a Foucaultian analysis with a phrase about how leper colonies later became mental asylums (this language was again linked to the text of *Morire di classe*).^11^ Were hospitals really meant to cure people, or simply to exclude them? The didactic voiceover then became a kind of lecture, citing progress in mental health reform the UK and France, complete with dates. The modern asylum system was linked directly to political and economic trends: ‘as bourgeois society is on the rise, it demands the marginalisation of socially unproductive elements’. What were asylums, Zavoli asked himself (and his audience)? ‘If the mentally ill are above all dangerous, should the rules on which these institutions are based work in line with this dangerousness or the illnesses from which they suffer?’ Like a priest, or a schoolteacher, Zavoli warned his viewers to be careful in their response. *They* were part of the reason that places like this existed, after all. He appeared to tick his viewers off. Now the voiceover became a sermon. ‘We need to free ourselves from much scepticism and just as much laziness in order to give a proper reply to this question.’

## Giving the patients a voice

In the penultimate and final acts of the film, the patients themselves came into sharp focus. They were given a voice. Zavoli introduced four one-to-one interviews as ‘a series of conversations which I filmed in Gorizia's asylum with the ill’. Extracts from four interviews were then shown, with the patients sitting on simple chairs in the gardens. Once again, Zavoli was out of shot.

In the first interview, a middle-aged man (who was not named, and who would turn up again in the final ‘scene’) related his experiences as a patient, and his loss of voting rights as he was interned in the *manicomio*. He then stressed the differences between the treatment offered to rich and to poor inmates. To illustrate this he told a long story about the treatment that had been offered to him under the previous regime, before Basaglia took over. The (ironic) conclusion/punchline to the story was similar to that of Basaglia earlier in the documentary: ‘the rich are not mad, at least from a giuridical point of view, while the poor are mad’ (Zavoli 1969b, 243). This patient was shot with a fixed camera, front on, with a cut later on to a profile shot. This technique, with some variations, would be repeated in all the interviews.

In the second dialogue/interview, a middle-aged woman (Carla Nardini) described how she had been treated in the ‘closed’ asylum. Hers was a story of institutionalized violence: ‘They tied me up, they beat me, they wanted to give me electro-shock treatment and I was terrified.’ This was ‘Carla’, an important figure in the Basaglian hospital, the secretary for many of the general meetings and an Auschwitz survivor who was interviewed early on in *L'istituzione negata*, in which she was described as ‘One of the best known and most listened-to patients in the hospital’ (Basaglia 1968, 24). Carla's interview was much more monosyllabic and staccato than the first conversation, with much shorter answers: ‘“What is a closed hospital?” “Closed? Prison.” “Did you have experience of the closed hospital?” “Yes.”’ Carla's interview touched on a series of issues: including her relationship with the outside world: ‘“How did the people in the outside world see you?” “Well, they know me. I am from Gorizia. After all, I am not a monster, you know….”’

**Figure 5:**
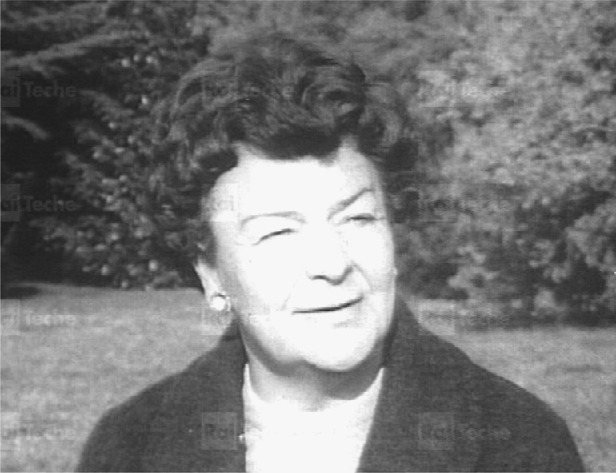
Interview with Carla, Gorizia. Rai-Teche.

**Figure 6:**
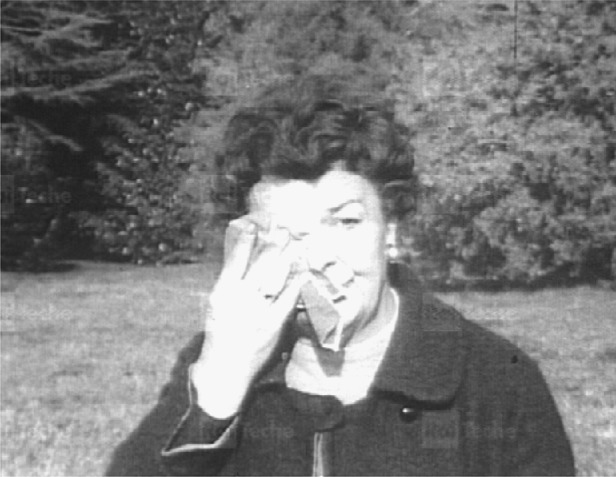
Carla crying. Rai-Teche.

Carla seemed both strong and fragile at the same time. She claimed that she ‘didn't care’ what people thought, but she also said that, in life, she was ‘all alone’. At one point she burst into tears, and her crying was captured and highlighted by the camera, as was her smoking (see Figures 5 and 6). It was, despite Zavoli's earlier raising of the issue, a voyeuristic moment. The camera did not flinch or move from her face. Pain and suffering was being used to create a spectacle – and to increase the TV audience.

In the third interview, with another middle-aged man, who was missing a few teeth, religious themes of redemption and sin re-emerged: ‘“Were you tied up? “Yes, but I am very religious. I suffered … but I also resisted….”’ The contrast, once again, was between a ‘closed’ and an ‘open’ hospital. This interviewee called the ‘closed’ hospital ‘a place of perdition’ where they treated people like ‘poor negroes’ and patients were tied to trees. Everything, however, had changed with the Basaglian revolution: ‘In the open hospital there is freedom, and a return to legality. We feel like men – we feel equal – and we are not afraid of our superiors, or the law.’ This patient said that he often went home, accompanied by nurses. He had been in the hospital for ten years.

**Figure 7:**
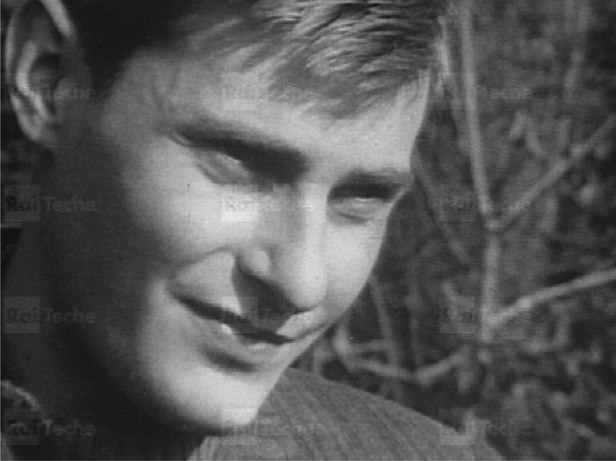
Pietro, interview. Rai-Teche.

**Figure 8:**
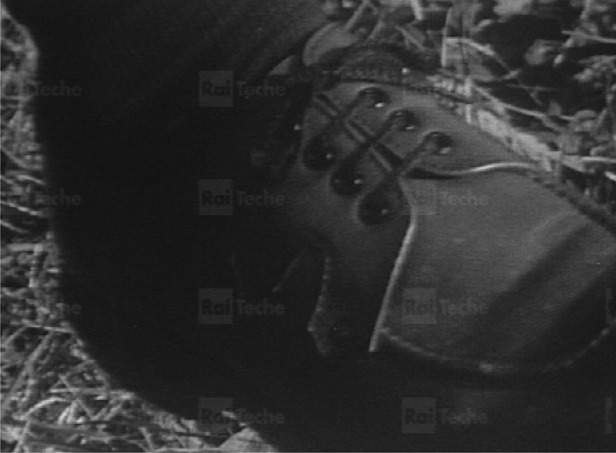
Pietro's foot. Rai-Teche.

The final patient (Pietro) was perhaps the most interesting of all. Good looking, young, blonde, fresh-faced, he was, in one sense, and certainly physically, almost the opposite of popular ideas and stereotypes about the ‘mad’ and their physical appearance. But he also had a slightly strange smile, and he shook his foot continually, something that was picked up by the camera, which zoomed in on his foot (see Figures 7 and 8). Pitrelli (2004, 74–75) argues that this shot created distance between the spectator and the interviewee.

The concentration on the feet of the patients (which happens twice in the documentary) might also have been a visual trope – taken in part from the photography of Luciano D'Alessandro (who published a series of photos of the hands of mental patients in a well-known and pioneering book which came out in 1965) and material in a previous documentary film, *1904* (Napolitano 1967). Zavoli had certainly seen that particular film as a clip from it was used in *I giardini di Abele* (D'Alessandro 1969; Randaccio 2013, 2, 4).

Pietro had some clear ideas about himself. He said that ‘a psychiatric hospital is a place which doesn't make sense, at the end of the day. In fact … *perhaps I am not ill*’ (1969b, 245, emphasis added). He said that he had first understood that he was not ill ‘in the old hospital’. Pietro wanted to get a job, and to have his rights returned to him (inmates in asylums were not allowed to vote). He claimed that he was already functioning in the outside world, where it was money that counted. The dialogue was marked by long pauses and close-ups. As the interview ended, the camera pulled back, leaving Pietro all alone on his chair, in the park of the asylum. The music then started up, again.

Zavoli used Pietro's interview to make a further series of points, as his voiceover returned, to interpret his words. The patients had been given a voice, but it was Zavoli who was in charge, and he would have the last word. First, Zavoli warned against a simplistic, social reading of mental illness.

The idea that it is just the poor who are mad is a fantasy. Both the poor and the rich can be mad, but the poor, in the face of illness, have no defence and are easily lost … and in a world which has no human values, those who cannot resist that ‘other’ prison – that world proudly inhabited by the sane – also get lost. Those who are able to criticize this reality are able to defend themselves. Pietro hasn't succeeded in doing so and he is now out in the world. And this is true of all those people in the Gardens of Abel – a motley crew who wander around in a confused way. (1969b, 248)

Basaglia's revolution had not resolved the problem of mental illness, or of the marginalization of the poor. There was much to be done. Outside, in the world of the ‘sane’, there was another kind of ‘prison’. These in-mates were an *Armata Brancaleone*, an Italian phrase linked to a famous comedy film of the 1960s, which ‘was used to refer to a mixed group, a collection of people with confused ideas who were highly disorganised’. The phrase might also be translated as ‘motley crew’ (Monicelli 1966).

**Figure 9:**
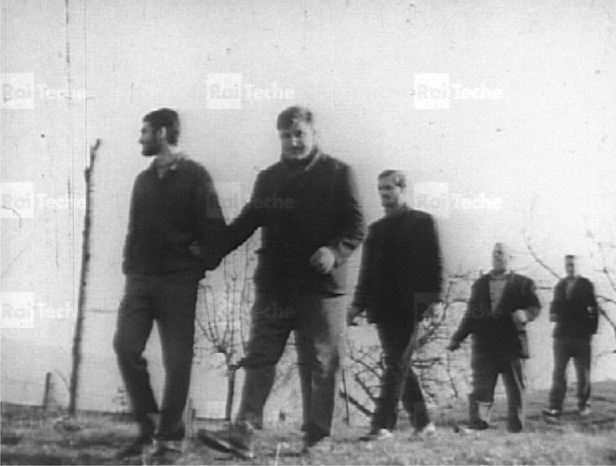
The ‘motley crew’ on the river-bank, Gorizia, final shots. Rai-Teche.

Then came the epilogue. We now saw this ‘motley crew’ of patients, volunteers and nurses walking along a river bank, outside of the hospital's grounds, with Mahler playing in the background. One of those walking was the first interviewee. The camera zoomed in on personal details, scuffed shoes, dirty clothing (see Figure 9). The film ended with this long march still ongoing. Where were these people going, if anywhere? What was their future? Did they have one at all?

## Conclusions

Zavoli's film was Basaglian and Zavolian. It discussed prejudice, it drew comparisons between concentration camps and asylums, it treated the patients as real people, it analysed social class. Moreover, it looked at stigma and at resistance to change. The film placed the viewer in a stark relationship with the inside of a psychiatric hospital, and with patients and ex-patients. For Zavoli, all this was *our* problem, *everybody's* problem, and it was also a social problem, not necessarily a medical one, not in the sense that social class ‘caused’ mental illness, but in the different way that people with the same illness were treated. Basaglia himself stated that he had ‘no idea’ what mental illness was, and he was much more interested in the ‘ill’ in any case. Onto these Basaglian traits, Zavoli added the ‘poetry’ that came from the combination of the images and the words being spoken, both in the voiceover and by the interviewees. He used and manipulated and commented upon the patient's words to make his own points about the lack of value in the ‘outside world’.

Finally, Zavoli gave the whole programme a religious edge and religious language – from the title onwards. It was as if Basaglia was carrying out a kind of mission. The programme was didactic, its message was rammed home, it was moralistic, and it was a history lesson, but it was also powerful and, in its own way, ‘revolutionary’, perhaps as much if not more so than *The Negated Institution* itself. Millions of Italians saw images that were radically different to any previous visual study of psychiatric hospitals. They saw Basaglia and heard his voice. Gorizia entered the living rooms of Italy.

But by the time *I giardini di Abele* was transmitted in early 1969, things had changed in Gorizia. Basaglia had left the city forever, along with many members of the original équipe. The film captured a moment in the asylum's history that was already part of the past. It was a historical document, even as it was transmitted.

This article has examined Zavoli's celebrated documentary *I giardini di Abele* (1969) in detail – looking at its visual features and its voiceover and dialogues. The links between the photobook *Morire di Classe*, which came out in 1969, have been noted and commented upon. Zavoli's film presented Basaglia's theories and examples of his practical work in Gorizia, but it also refracted and adapted those ideas, and that practical work, to Zavoli's own ideas and his own message. This was a film about Basaglia and Gorizia, but it was above all a film ‘by Sergio Zavoli’. This article has presented the first detailed analysis of this well-known and oft-cited film, and the first detailed reading of its contents, as well as its context. As for the effects of the film on popular opinion, this has often been assumed in the secondary literature, but in reality was almost certainly complicated and contradictory. In many ways, it is impossible to say what the real effects of this documentary were in 1969 and since. Given this, it is important to return to the document itself and analyse what is really there – not merely what has been picked out in a largely celebratory and superficial way by most of those who have written about *I giardini di Abele*.

## Footnotes

^ 1^ The full version of the film can be seen online at http://www.deistituzionalizzazione-trieste.it/videogallery/VideoSourceITA.html. This article is based on the original transmission version, obtained from the RAI archive. There are numerous truncated versions available on the web. Copyright issues often mean that the opening scene has been cut.

^ 2^ There are various published versions of the script for *I giardini di Abele*, many of which differ from that which was actually transmitted in 1969. The quotes in this article are based, unless otherwise stated, on the film itself (Archivio RAI, Teca D4245/B). See, for edited versions of the script, Zavoli (1969, 237–248, 2002, 363–378. 2005).

^ 3^ These were not terms widely used by the Basaglians themselves.

^ 4^ See the documentary *Un giorno al manicomio* (1961) shot in Rome's asylum (http://www.rai.tv/dl/RaiTV/programmi/media/ContentItem-5bb346cc-4926-417c-a066-d31d37ff720f.html) and the comparison made with *I giardini di Abele* in a recent TV history programme (https://www.youtube.com/watch?v = gYxiwe7QpRs).

^ 5^ This citation appeared on the back cover of the first collective book produced by the équipe about Gorizia, *Che cos'è la psichiatria?* (Basaglia 1967).

^ 6^ The dramatic clip of a patient being forcibly restrained by two nurses was taken from Riccardo Napolitano's (1967) documentary, *1904, n. 36*. It is not clear if this was a real incident, however, or a ‘reconstruction’ using actors.

^ 7^ The preparations would appear to have been going on for a long time. There are signs that journalists from TV7 were in Gorizia in 1966. Zavoli got to know Basaglia well, and was a visitor to his flat in Gorizia and to the hospital itself in 1967–1968. The script was seen by Basaglia before transmission, and it is clear that extensive negotiation took place over its content.

^ 8^ Archivio Basaglia (no date, but 1968).

^ 9^ For a taste of this chaos, see the film shot by a Finnish documentary crew in 1968, *La favola del serpente* (http://www.youtube.com/watch?v = tkm4rXqjWIs and http://www.youtube.com/watch?v = 8No0yr0NsLM).

^10^ Symphony n. 1 ‘Titan’, II movement.

^11^ One photo from the film was taken by Berengo Gardin, but did not appear in *Morire di Classe*, so it is clear that Zavoli was given access to the range of photos from which the selection was made by the Basaglias for that book.
